# Whole-Genome Sequencing in Newborn Screening—Attitudes and Opinions of Bulgarian Pediatricians and Geneticists

**DOI:** 10.3389/fpubh.2017.00308

**Published:** 2017-11-20

**Authors:** Georgi Iskrov, Stefan Ivanov, Stephen Wrenn, Rumen Stefanov

**Affiliations:** ^1^Department of Social Medicine and Public Health, Faculty of Public Health, Medical University of Plovdiv, Plovdiv, Bulgaria; ^2^Institute for Rare Diseases, Plovdiv, Bulgaria

**Keywords:** newborn screening, genetic screening, whole-genome sequencing, public health, rare diseases, European reference networks

## Abstract

**Objective:**

The aim of this study was to assess the attitudes and opinions on the potential use of whole-genome sequencing (WGS) in conjunction with the traditional newborn screening (NBS). We conducted an online survey among pediatricians and geneticists from Bulgaria. The study was based on the concept of non-selective WGS for all newborns and analysis of all genes.

**Results/conclusion:**

In total, 120 out of 299 invited participants completed the survey, with an overall response rate of 40.1%. While half of the pediatricians surveyed supported population-based non-selective WGS in NBS, 65.2% of the geneticists expressed concerns. Most participants underlined that ethical issues were as important as medical ones and called for a stricter protection of affected individuals against any abuse of their personal data. Extensive genetic counseling and psychological support to families were mentioned as key elements in this potential activity. Nevertheless, both pediatricians and geneticists considered that NBS in Bulgaria could be further developed, with selective WGS being suggested as a potential option. While non-selective WGS for all newborns is not currently perceived as feasible, pediatricians and geneticists do believe that selective WGS could strengthen current NBS programs. Cross-border project collaborations may set the stage for generating experience and evidence on these complex issues.

## Introduction

Newborn screening (NBS) is a public health service aimed at detecting apparently healthy infants with severe congenital disorders, for which there are available cost-effective identification and effective treatment. NBS is considered to be the longest running and most successful population screening activity worldwide (1). Many inborn conditions are potential candidates for NBS. World Health Organization’s Wilson–Jungner criteria are regarded as a benchmark that disorders need to meet in order to be included in an NBS panel. These indicators cover a range of issues, including knowledge on the disease, treatment, test validity, and costs (2).

Whole-genome sequencing (WGS) is viewed as a major vehicle for translating genetic and genomic advances into population health gains. The debate as to whether to include WGS in NBS is currently taking place in many jurisdictions (3–5). There are, however, considerable concerns among health-care practitioners, including limited benefit of WGS for most healthy people in the general population, lack of expertise among non-genetic health-care providers, potential negative implications for society and scarce economic resources (6–8). It is clear that there is a gap between what is technically possible and the clinical services available. The rate at which new disease genes are being identified is out-pacing the ability of medical professionals and health authorities to assess the potential risks and benefits of introducing WGS in NBS (9).

In Bulgaria, NBS started in 1979 and included testing for phenylketonuria and galactosemia. The latter was removed from the panel in 1989 due to lack of effectiveness. A pilot NBS for congenital hypothyroidism was carried out in 1993–1999 as a joint Swiss-Bulgarian research project. The current NBS panel in Bulgaria includes phenylketonuria, congenital hypothyroidism, and congenital adrenal hyperplasia, and it is mandated by law and publicly funded (10, 11).

### Objective

The purpose of this study was to assess the attitudes and opinions of pediatricians and geneticists on the potential use of WGS for NBS in Bulgaria.

## Materials and Methods

### Definitions

This study is based on the concept of non-selective WGS for all newborns and analysis of all genes. This definition of WGS was presented to all study participants and included in the subsequent discussion of the results.

### Study Design

Scope and format of the study were based on previous experiences by Ulm et al. and Joseph et al., as well as on the outcomes of an evaluation study of NBS for rare disorders in the Member States of the European Union (EU) in 2012 (5, 12, 13). The survey questionnaire consisted of 20 questions. Each question contained a free text field for providing additional information, if desired. Before starting the questionnaire, participants were given brief definitions of WGS and NBS. The questionnaire was piloted among a small group of medical professionals for input regarding clarity. The study was conducted through an online survey in March–April, 2017 in Bulgaria.

Ethical approval was not required for this study in accordance with the national and institutional guidelines. The survey was sociological from a methodological point of view, with no clinical research. No personal data were saved or analyzed.

### Study Participants

Study participants included geneticists and pediatricians from Bulgaria. Selection of these target groups was based on the state-of-art of NBS in Bulgaria, as well as the perspectives of implementing WGS in the country. These medical professionals are currently involved in NBS and in the subsequent diagnostic confirmation, treatment, and follow-up of detected cases. Geneticists and pediatricians would be required to be actively engaged if WGS was incorporated into NBS in Bulgaria.

A convenient sample of participants was recruited from the membership of the Bulgarian Society of Human Genetics and Genomics and the Bulgarian Pediatric Association with publicly available email addresses. A total of 299 individuals were contacted by email to participate in the survey with an invitation letter describing the study. No incentives for participation were provided.

### Data Analysis

Descriptive statistics were applied. Comparisons were made between different demographic variables to determine if they were associated with specific outcomes. Analyses specifically focused on the differences between pediatricians and geneticists. Chi-square and Fisher’s exact tests, as well as Mann–Whitney *U*-test were used to compare these two groups. Statistical significance was considered if the *p*-value was less than 0.05. Data were analyzed using SPSS (version 11.5; SPSS, Inc., Chicago, IL, USA).

## Results

### Profile of Survey Respondents

In total, 120 out of 299 invited participants completed the survey, with an overall response rate of 40.1%. Seventeen respondents indicated no medical specialty, so they were excluded from further analysis. Mean professional experience of the participants was 21 years with 54.4% of them having a Ph.D. degree (Table 1). The majority (89.3%) indicated a clinical role in a primary professional position, with 80 having a specialty of pediatrics (general pediatrics and/or profiled pediatrics) and 23 a specialty of genetics. Half of the respondents (53.4%) reported having a second specialty. Fifty-one pediatricians had a second, profiled specialty, as did four geneticists, who had a second specialty of pediatrics. Nevertheless, the latter were classified as geneticists for the purpose of cross-comparison. The groups of pediatricians and geneticists were found to be similar in terms of sex, age and professional experience (Table 1).

**Table 1:** Profile of survey respondents.

Profile characteristic	Overall, % (*n*)	Pediatricians, % (*n*)	Geneticists, % (*n*)	*p*-Value
**Gender**				
Male, % (*n*)	17.48% (18)	16.25% (13)	21.74% (5)	>0.05
Female, % (*n*)	82.52% (85)	83.75% (67)	78.26% (18)	
Age, years (±SD)	48.9 ± 10.6	49.13 ± 10.83	48.17 ± 9.81	>0.05

**Highest degree**				
M.Sc., % (*n*)	34.95% (36)	40.00% (32)	17.39% (4)	>0.05
Ph.D., % (*n*)	54.37% (56)	50.00% (40)	69.57% (16)	
D.Sc., % (*n*)	10.68% (11)	10.00% (8)	13.04% (3)	
Second medical specialty, % (*n*)	53.40% (55)	63.75% (51)	17.39% (4)	<0.0001
Professional experience, years (±SD)	21.1 ± 11.8	21.65 ± 12.01	19.39 ± 11.21	>0.05

**Sector (>50% of the time)**				
Public, % (*n*)	73.79% (76)	70.00% (56)	86.96% (20)	>0.05
Private, % (*n*)	8.74% (9)	8.75% (7)	8.69% (2)	
Equally, % (*n*)	17.47% (18)	21.25% (17)	4.35% (1)	

**Main professional role**				
Administration, % (*n*)	0.97% (1)	0.00% (0)	4.35% (1)	<0.0001
Diagnosis and treatment, % (*n*)	89.32% (92)	96.25% (77)	65.22% (15)	
Research, % (*n*)	3.88% (4)	2.50% (2)	8.69% (2)	
Teaching, % (*n*)	5.83% (6)	1.25% (1)	21.74% (5)	

### Knowledge and Awareness of NBS and WGS

**Figure 1:**
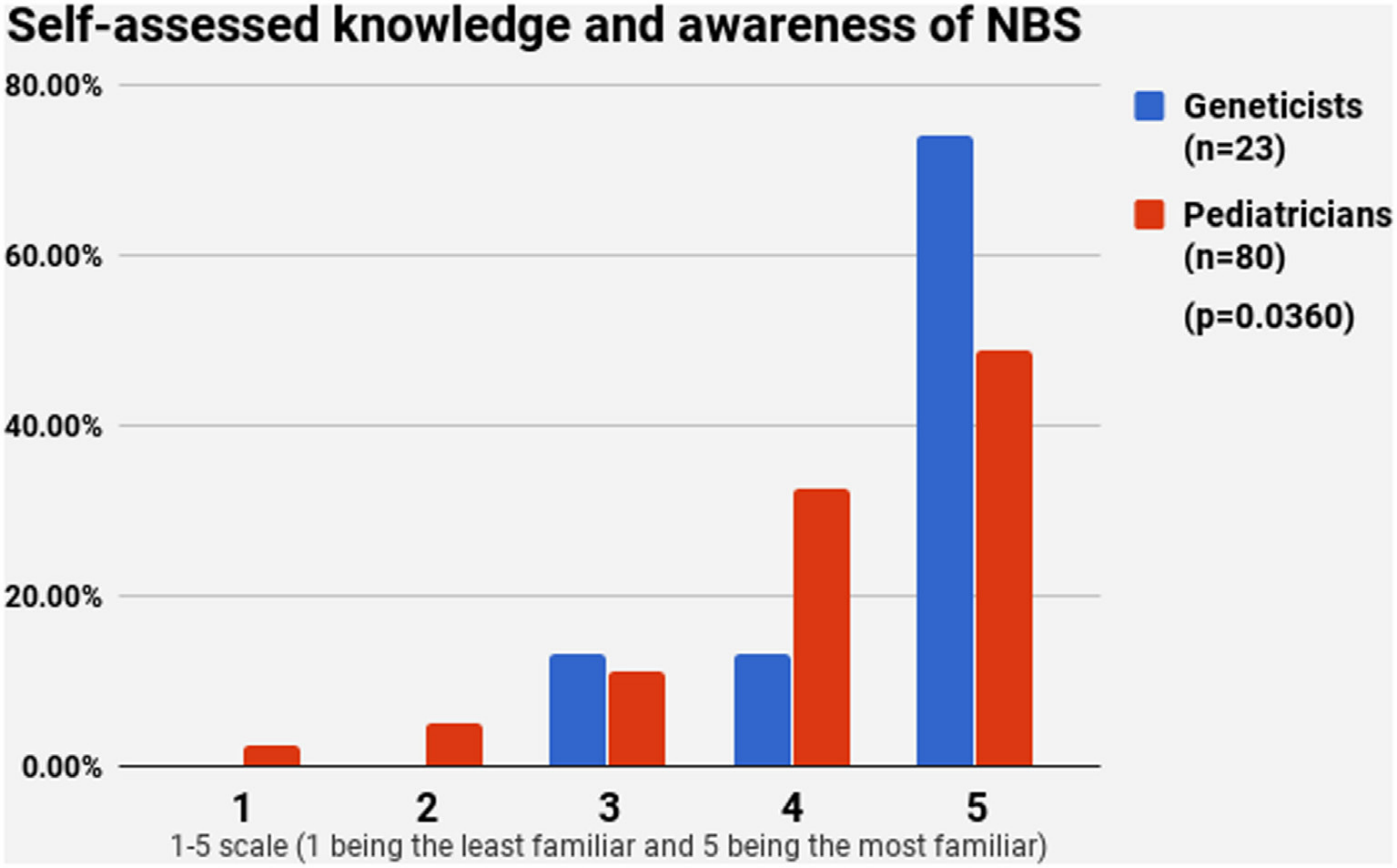
Self-assessed knowledge and awareness of newborn screening.

**Figure 2:**
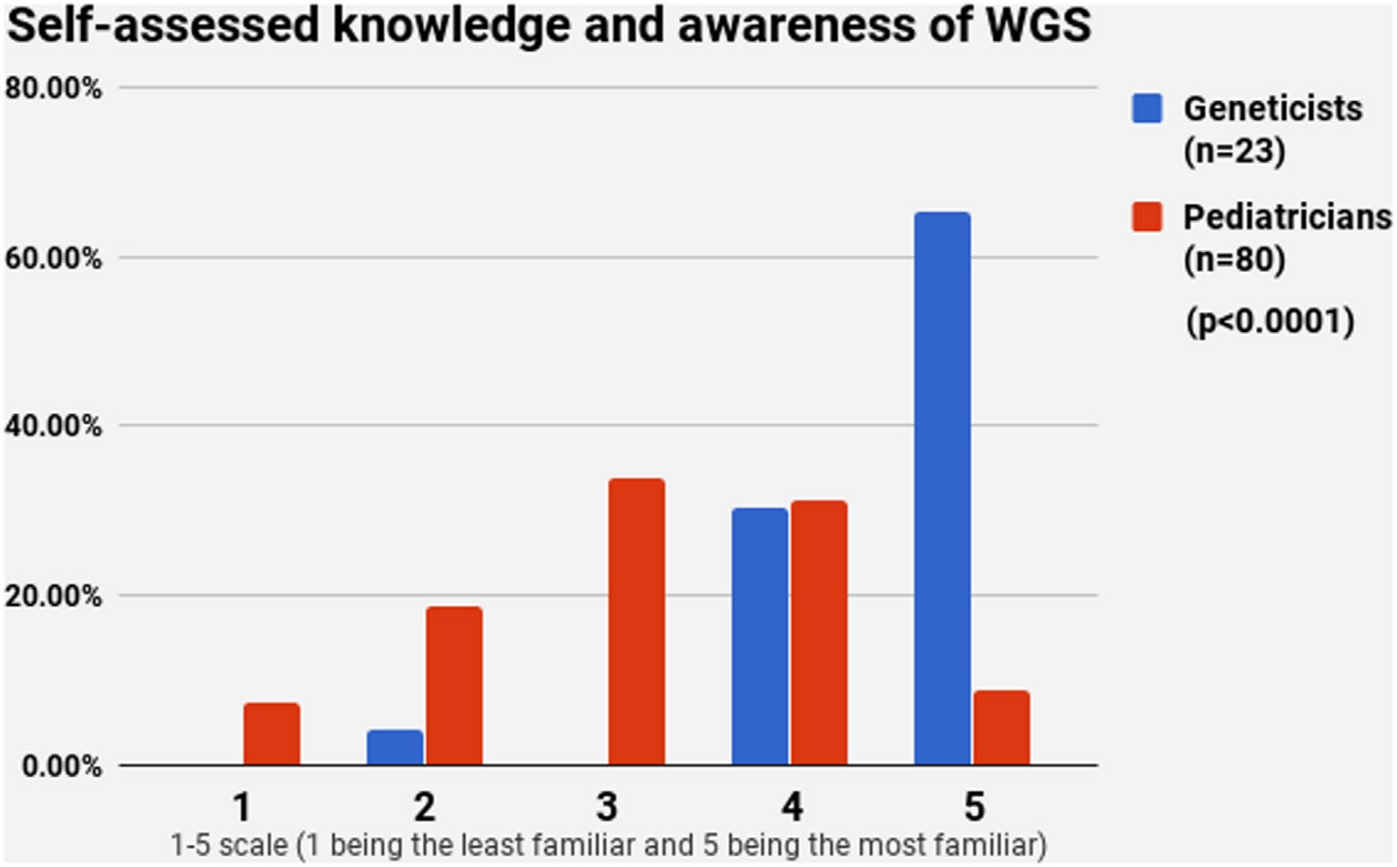
Self-assessed knowledge and awareness of whole-genome sequencing.

**Figure 3:**
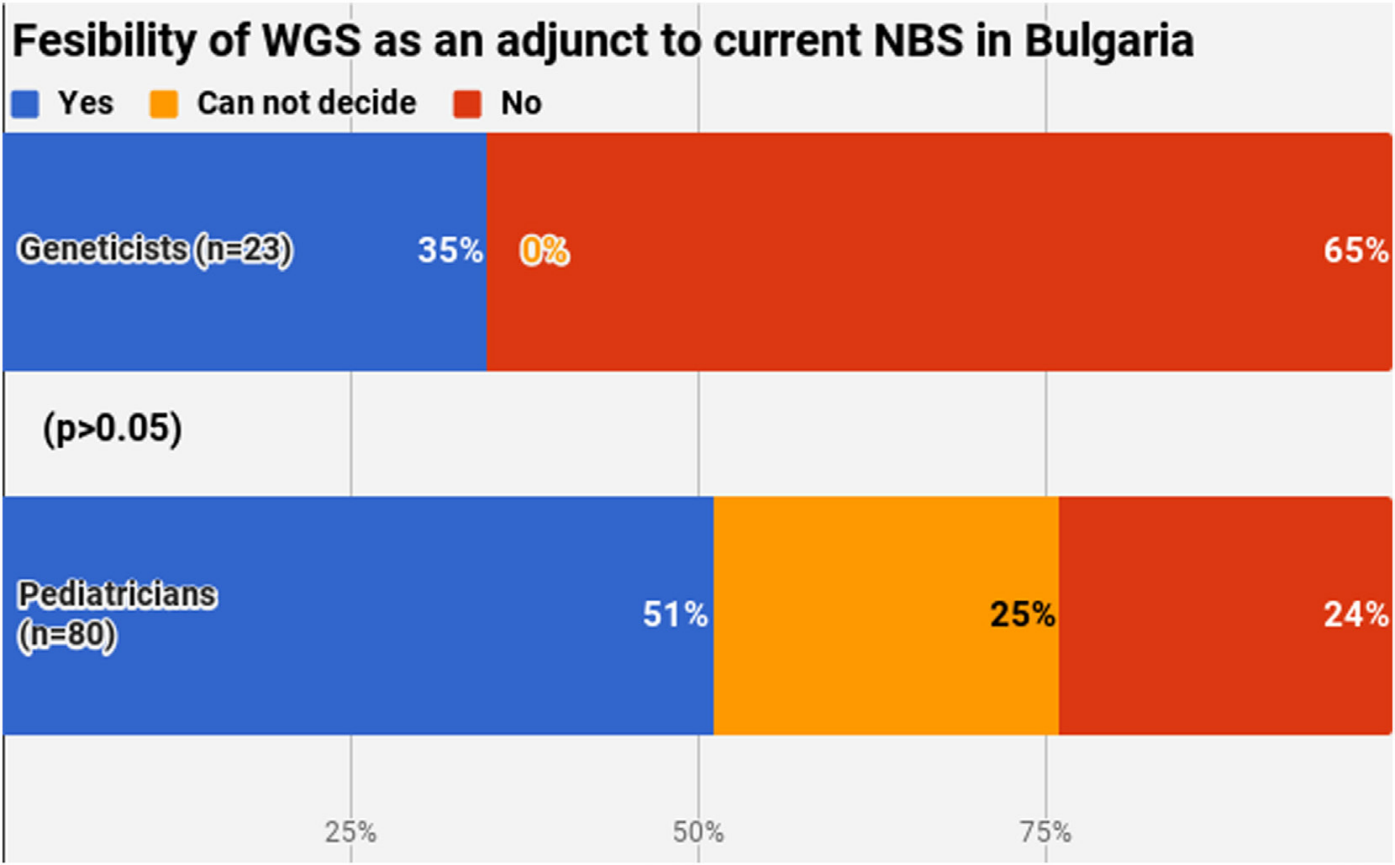
Fesibility of whole-genome sequencing as an adjunct to current newborn screening in Bulgaria.

Half of the participants (54.4%) indicated a maximum level of knowledge on NBS on a self-assessment scale, while only 21.4% reported highest level of awareness on WGS. The difference between pediatricians and geneticists was statistically signficant in both cases (Figures 1 and 2). This was especially pronounced in regard to WGS, with 15 out of 23 geneticists declaring highest level of knowledge in comparison to only 7 out of 80 pediatricians. The split in self-assessed knowledge of NBS and WGS reflected on the question whether WGS could be implemented as an adjunct to NBS in Bulgaria (Figure 3). Whereas 25.0% (*n* = 20) of the pediatricians responded, they could not decide about this issue, all geneticists clearly indicated their opinion here. Only 34.8% (*n* = 8) of them supported the feasibility of WGS in NBS, while the rest opposed it (65.2%, *n* = 15). On the other side, pediatricians demonstrated enthusiasm, with 51.2% (*n* = 41) supporting the idea versus 23.8% (*n* = 19) being against. However, this disagreement was not statistically significant (*p* > 0.05).

Geneticists used the free text field to further point out their concerns (Table 2). The fact that most of the WGS data could not be directly linked to a specific role for the individual’s phenotype and development, thus generating lots of uncertainty and ambiguity, was emphasized. A large number of both groups raised the ethical issues that may occur, including the possibility of WGS data being used to the detriment of the individual. While high costs were rarely discussed, many participants mentioned that at this moment no country routinely used WGS as a part of NBS. Nevertheless, both pediatricians and geneticists considered that NBS in Bulgaria could be further developed, with selective WGS being suggested as a potential option by a large number of respondents.

**Table 2:** Respondents’ additional comments on regulatory settings and organizational issues.

Survey question	Comment (P = pediatrician, G = geneticist)
Do you consider feasible implementing whole-genome sequencing (WGS) as an adjunct to current newborn screening (NBS) in Bulgaria?	G1: *“I do not think that our society is ready to accept and interpret all the information obtained through WGS. There are numerous ethical issues and problems to consider as well.”*
	G2: *“WGS is still too expensive. This could be only suitable for selective screening—under specific rules for certain indications and populations.”*
	P1: *“Currently, WGS is not routinely applied along NBS in any country or jurisdiction. However, I definitely believe NBS in Bulgaria could be greatly expanded.”*
	P2: *“In this case, WGS would provide answers to non-existent questions, which could be extremely damaging for the patients and their families from a psychological point of view.”*
	G5: *“Having in mind that at this point the majority of WGS information can not be linked to a specific clinical manifestation, WGS is not feasible in the context of mass NGS. Furthermore, this may violate individual’s rights, as we would explore the genome of a person without his/her consent.”*
	G6: *“There are too many ethical issues to be addressed first.”*
	P4: *“I think that funds should be directed first to expand the current NBS panel, as well as to train specialists in WGS.”*
	P5: *“This is a very expensive project with no clear health benefits. WGS could be only feasible in some very specific conditions and indications.”*
	P6: *“This could be feasible in cases with a family history of genetic disorders.”*
	G7: *“I believe that a more precise target population would be more reasonable in this situation. A research project like “100,000 Genomes Project” could be much more meaningful.”*
In case of WGS being implemented as an adjunct to NBS in Bulgaria, what do you think is the most appropriate way to regulate this process?	G1: *“Genetic counseling should be a mandatory prerequisite for that.”*
	G2: *“I think that selective screening is the only reasonable option. WGS should be an adjunct diagnostic tool for specific conditions in the neonatal period.”*
	G5: *“WGS should be optional at the parents’ request and only after genetic counseling.”*
	G8: *“I would accept selective WGS in cases of pediatric onset conditions. When the individual attains legal age, he/she could decide what genetic tests to undergo. Patient has the right to decide what genetic information he/she carries. WGS in NBS eventually takes away this right from the individual.”*
	G9: *“Only as a selective screening for predetermined conditions.”*
	G10: *“I think it is too early to discuss the application of WGS as a screening method. I consider selective genome sequencing for known mutations as the only realistic option at this moment.”*
In case of WGS being implemented as an adjunct to NBS in Bulgaria, what do you think is the most appropriate way to regulate additional research with collected and processed anonymized samples?	G1: *“In case of WGS being implemented as an adjunct to NBS in Bulgaria, anonymized samples should be a priori used for additional research activities.”*
In case of WGS being implemented as an adjunct to NBS in Bulgaria, what types of WGS results should be disclosed to parents?	G2: *“Interpretation of information on carrier status and predispositions should be given to the person after attaining legal age.”*
	G11: *“This issue needs a detailed legal framework to define standard rules and procedures.”*
	G8: *“This is not feasible. I would like to see a fellow geneticist who is going to explain all genes, as well as a patient who is going to listen to all this information. And then, to see a patient and his/her family who are going to come every 5 years to get updated on the current advances of genome sequencing. This is not possible. Parents should be informed on all identifiable pediatric onset conditions (both treatable and non-treatable). Additionally, they could choose whether to get information about predisposition to pediatric conditions. Anything else could be provided to the person after attaining legal age if he/she wants to.”*
	G6: *“Parents should decide what information on pediatric conditions they want to know. After attaining legal age, the individual could decide what other information to know.”*
	G12: *“Parents should be only informed about treatable conditions.”*
	G10: *“Parents should decide in advance what type of results to be disclosed. This should follow some standardized procedures.”*
In case of WGS being implemented apart from NBS in Bulgaria, what is the best time to carry out this activity?	G2: *“It is not realistic to conduct mass WGS. This is only reasonable during the neonatal period in cases of proven clinical benefit.”*
	G11: *“Only as a supportive diagnostic tool. Otherwise, after attaining legal age.”*
	G5: *“Mass WGS screening is not cost-effective. Selective WGS screening could be, however, beneficial under specific conditions and indications.”*
In case of WGS being implemented as an adjunct to NBS in Bulgaria, what do you think is the most appropriate source to fund this activity?	G2: *“Funding through research projects and subsequent public funding if justified is the best option. However, I believe that it is much more reasonable to elaborate a list of specific conditions with high clinical and economic burden and to conduct selective genome screening only for them.”*
Final overall comments	G3: *“WGS is the basis of predictive medicine. Predictive medicine is not a therapeutic but a prophylactic approach. Predictive medicine, along with prevention medicine and personalized medicine is the future of medicine and healthcare.”*
	P3: *“Introduction of new screening programs should be a result of broad discussion and consensus, justified by clinical and economic evidence.”*
	G4: *“Bulgarian stakeholders need to study first the experience of other countries.”*
	P2: *“This survey is right on time, because there are more and more speculations on this issue.”*
	P4: *“I think we should first take care of families with a history of genetic diseases and provide prenatal diagnosis in these cases. Mass WGS in NBS is utopia. WGS in general population is not cost-effective. Furthermore, information about genetic markers may cause unnecessary psychological burden to patients and their families.”*
	P5: *“Funding is the main problem here. No country can afford WGS in mass NBS.”*
	G13: *“I do support genome research. I very much hope that the benefits would outnumber the risks. But as a physician, I stay behind the patient’s right of autonomy and right to refuse such testing and information.”*
	G14: *“I do not consider WGS in NBS to be an important issue at the present moment. WGS in mass NBS is not feasible.”*
	P6: *“This could lead to serious psychological problems for the child and the family. They could be subject of discrimination.”*
	G12: *“WGS has its own potential in medicine. But at this moment, this will generate more questions than answers. In the context of assessing and predicting individual’s health, we are not ready to interpret correctly large-scale data like these.”*
	P7: *“WGS should not be the future of medicine, WGS must be the present.”*
	P8: *“In my view, WGS is still too costly to be used extensively. Its application should be carried out in the neonatal period in case of suspicion of a genetic disease (clinical symptoms or high family risk). The decision must be discussed with the parents and WGS should take place only upon informed consent.”*
	P9: *“I think this is a very good idea for a survey. Nevertheless, I believe for the time being we need more precise genetic diagnosis for certain severe chronic diseases where treatment depends on the genetic mutation. At a later stage, this could be done along NBS and provide an individual prognosis for each child. But for now it brings me ideas of fiction novels with many extreme, undesirable outcomes.”*
	G5: *“Only a very small portion of WGS results could be categorically interpreted at this moment. The rest falls in the so called gray zone with no use in clinical practice. Of course, our knowledge and understanding increase by each new case studied. But this should be done within research projects, not along mass NBS. My opinion is completely different about genome sequencing for known mutations, which I strongly support.”*

**Figure 4:**
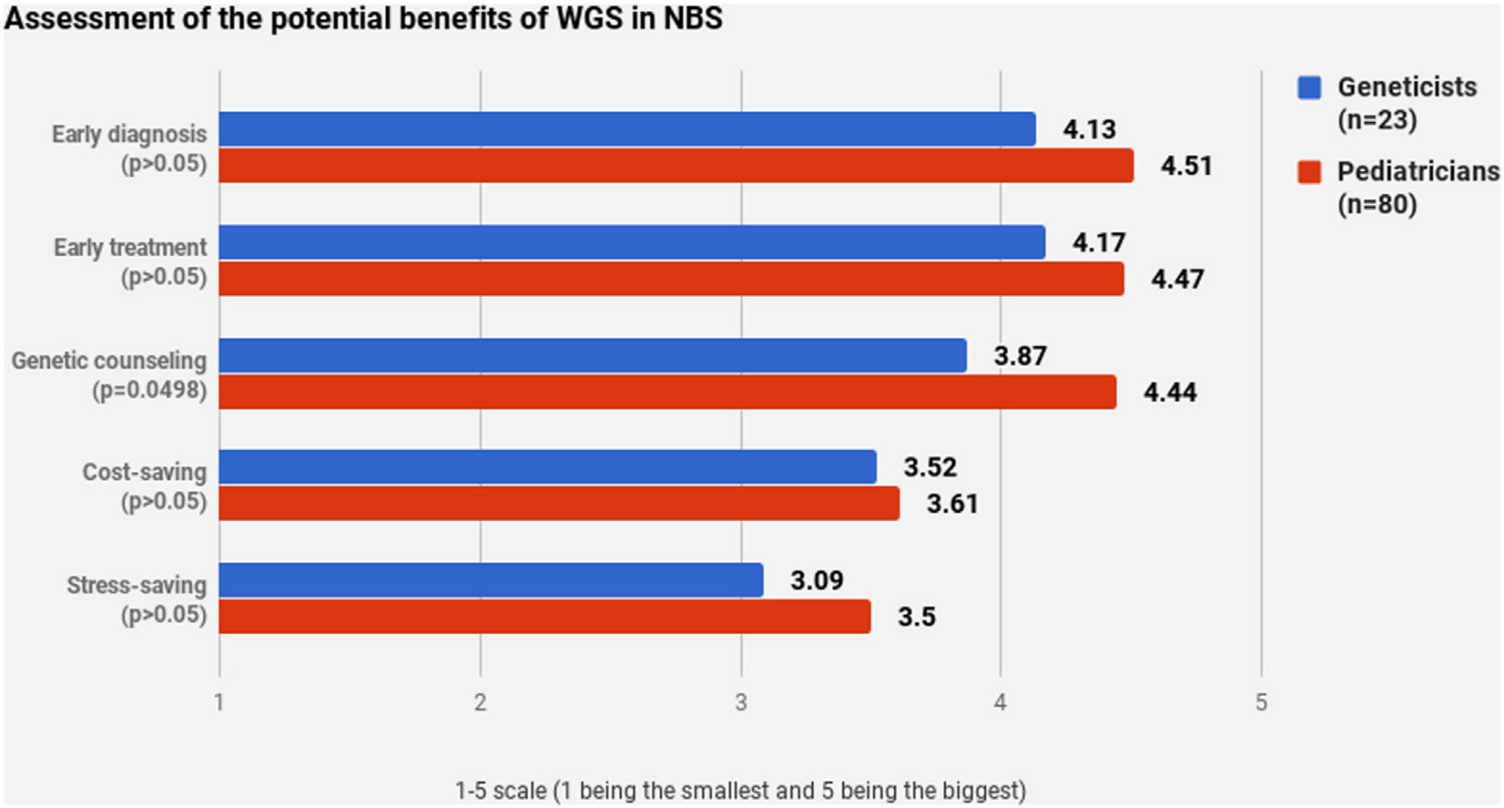
Assessment of the potential benefits of whole-genome sequencing in newborn screening.

**Figure 5:**
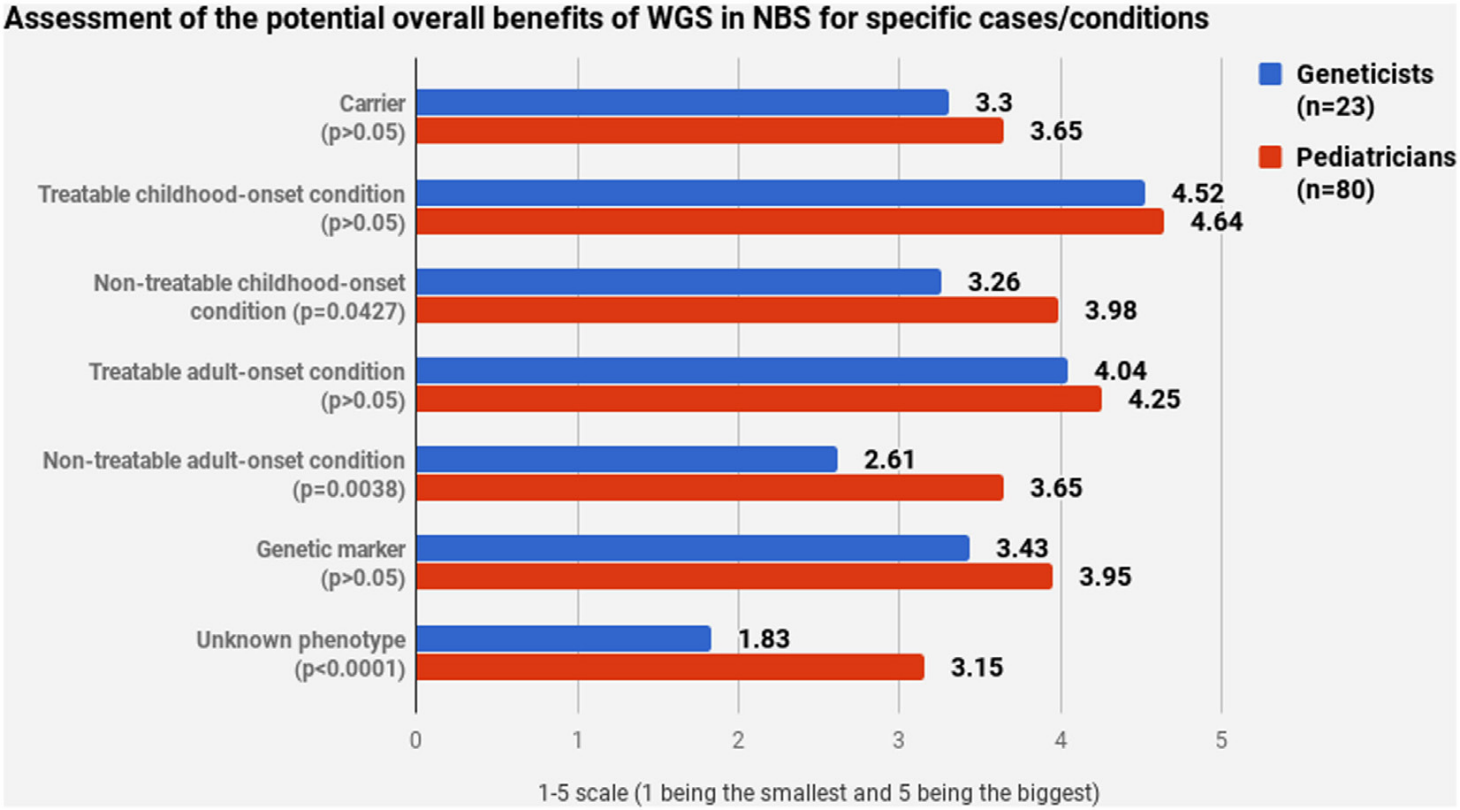
Assessment of the potential overall benefits of whole-genome sequencing in newborn screening for specific cases/conditions.

Pediatricians and geneticists largely agreed on their assessment of WGS’s potential benefits (Figures 4 and 5). WGS was seen as especially helpful for early diagnosis and treatment. Nevertheless, a stable scoring pattern was observed with pediatricians giving higher ratings than geneticists. The same outcome was observed when respondents were asked to evaluate WGS’s overall benefits in specific cases. The two groups agreed on the benefits of WGS in scenarios that are medically actionable, specifically for detection of treatable childhood-onset and treatable adult-onset conditions, as well as carriers and genetic markers. Statistically significant disagreement occurred in cases that are not medically actionable—childhood-onset non-treatable (*p* = 0.0427) and adult-onset non-treatable (*p* = 0.0038) conditions, as well as unknown phenotypes (*p* < 0.0001) (Figure 5).

### Regulatory Settings and Organizational Issues

Study participants were asked about their opinion on regulatory settings and organizational issues if WGS is hypothetically introduced as a part of NBS in Bulgaria. Not a single geneticist supported the idea of making WGS mandatory, while 16 pediatricians expressed support (Table 3). Across both groups, 44 respondents (42.7%) believed that WGS should not be mandatory and must be available only upon parental request. While the majority of pediatricians indicated that WGS should be a mandatory or highly recommended service in this hypothetical case, most geneticists supported its use only upon request or in the presence of specific symptoms (*p* = 0.0197). This position was further reflected in the free text comments (Table 2). A geneticist pointed out that neither the projected high costs nor the inability to correctly interpret all WGS results are the main obstacle. It was the impact on the individual affected and his/her legitimate right not to know what genetic information he/she is carrying. The respondent stressed that WGS in NBS is effectively taking this power away from the person with no clear benefits in return.

**Table 3:** Regulatory settings and organizational issues.

Regulatory setting/organizational issue	Overall, % (*n*)	Pediatricians, % (*n*)	Geneticists, % (*n*)	*p*-Value
**Legal mandate of whole-genome sequencing (WGS) in newborn screening (NBS)**				
Mandatory	15.53% (16)	20.00% (16)	0.00% (0)	0.0197
Optional, but highly recommended	33.01% (34)	35.00% (28)	26.09% (6)	
Upon parental request	42.72% (44)	42.50% (34)	43.48% (10)	
Other	8.74% (9)	2.50% (2)	30.43% (7)	

**Funding of WGS in NBS**				
Public	14.56% (15)	17.50% (14)	4.35% (1)	>0.05
Private	1.94% (2)	1.25% (1)	4.35% (1)	
Research project	4.85% (5)	6.25% (5)	0.00% (0)	
Research project and subsequent public funding if justified	78.64% (81)	75.00% (60)	91.30% (21)	

**Disclosure of WGS results**				
All results	64.08% (66)	75.00% (60)	26.09% (6)	<0.0001
Upon decision by the physician	6.80% (7)	3.75% (3)	17.39% (4)	
Upon decision by the family	21.36% (22)	17.50% (14)	34.78% (8)	
Upon decision by the physician (further decision by the person after attaining legal age)	7.77% (8)	3.75% (3)	21.74% (5)	

**Additional research of WGS samples**				
No additional research conducted	4.85% (5)	6.25% (5)	0.00% (0)	>0.05
Consent required	69.90% (72)	71.25% (57)	65.22% (15)	
Assumed consent	2.91% (3)	2.50% (2)	4.35% (1)	
No consent required	22.33% (23)	20.00% (16)	30.43% (7)	

**Timing of WGS if apart from NBS**				
At birth	65.05% (67)	70.00% (56)	47.83% (11)	>0.05
At age of 0–6	11.65% (12)	13.75% (11)	4.35% (1)	
After attaining legal age	2.91% (3)	0.00% (0)	13.04% (3)	
In case of specific symptoms	20.29% (21)	16.25% (13)	34.78% (8)	

A strong consensus was observed on the funding issues. Eighty-one respondents (78.6%) agreed that WGS should be initially funded through research projects and subsequently by public funds if substantial public health benefits are demonstrated (Table 3). There was no clear consensus among pediatricians and geneticists regarding the disclosure of results. While 75.5% (*n* = 60) of pediatricians believed that all WGS results should be disclosed, no option was supported by a majority of geneticists (*p* < 0.0001). Only 26.0% (*n* = 6) of them indicated that all results should be disclosed. Furthermore, many of the geneticists surveyed explained in the free text field that this process should be a result of extensive genetic counseling (Table 2). With regards to the use of WGS data for additional research purposes, a general consensus was reached, as 69.9% (*n* = 72) declared that additional research was only appropriate if informed consent was given. In case of WGS being implemented apart from NBS in Bulgaria, this activity was found to be most appropriate to conduct at birth, 65.0% (*n* = 67), and in case of specific symptoms, 20.4% (*n* = 21), with 70.0% of pediatricians and 47.8% of geneticists supporting the first option (*p* > 0.05).

**Figure 6:**
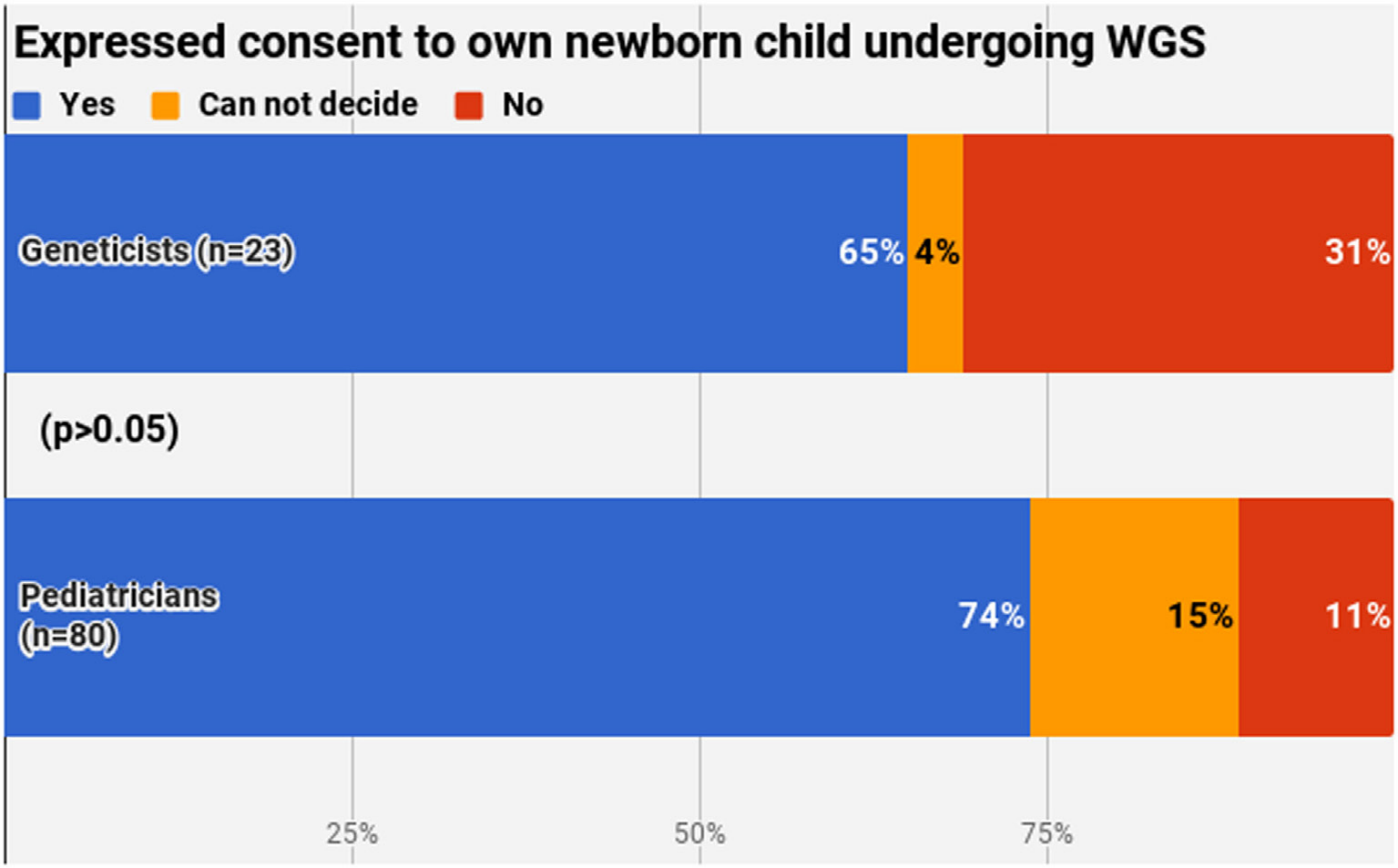
Expressed consent to own newborn child undergoing whole-genome sequencing.

The final section of the questionnaire aimed to describe the personal attitude of the respondents. A clear majority (71.8%, *n* = 74) would consent to their own newborn undergoing WGS, with no significant difference between pediatricians and geneticists (*p* > 0.05) (Figure 6). Most common reasons for yes included the benefits of this procedure and WGS seen as the future of medicine. Most common reasons for no were the absence of a clear need for this service and the possible ethical issues arising.

## Discussion

### General Attitudes on WGS Implementation Alongside NBS

There is an ongoing debate regarding the role of WGS in NBS, but large-scale research activities, such as the USA’s Precision Medicine Initiative and the UK’s 100,000 genomes project, could lead to a reconsideration of WGS in all fields of medicine and health care. Respondents in our study admitted that WGS in NBS is hard to imagine in a small country with limited resources like Bulgaria, before this activity becomes commonly accepted and routinely applied in bigger jurisdictions. A geneticist commented that he/she does not see WGS in NBS being implemented in Bulgaria in the next 10 years. Nevertheless, all participants agreed that such a survey is needed in order to set up the stage for further research, as well as to dismiss uncertainty and speculations. As the issue of WGS in NBS is going to be raised more and more, the insights from our study could be highly beneficial at regional and European levels.

Previous studies extensively presented geneticists’, genetic counselors’, and parents’ perspectives, as well as general public views on WGS in NBS (5, 12, 14–17). To our best knowledge, our survey is the first to explore and cross-compare opinions of pediatricians and geneticists. This fills a substantial knowledge gap. There is no doubt that geneticists have a leading role in WGS. However, when evaluating the prospects of WGS in conjunction with NBS, pediatricians represent an important stakeholder whose views must be considered and taken into account. The opinion of pediatricians is crucial, given the fact that they would be exclusively involved in treatment and follow-up of all potential patients.

The demonstrated lack of clear consensus on whether WGS should be incorporated into NBS illustrates that there is a need for discussion and collaboration among all stakeholders before any major changes are being implemented. A recent study indicated that the vast majority of the American geneticists feel that WGS should not be currently used in NBS, and that if it to be used, it should not be mandatory (12). This attitude was greatly shared by geneticists from our sample. Pediatricians, however, had a rather different point of view. It is unclear if this difference is a result of the different level of expertise in WGS or it is because of the unique role of pediatricians in the treatment and follow-up of children. In our study, this specific group of respondents saw WGS benefits more positively in cases of non-treatable conditions than geneticists. So, a desire to manage the at-risk children might have been the basis for the discriminatory viewpoint.

### Crucial Points for Consideration of WGS Implementation Alongside NBS

Ethical issues were the most commonly cited concerns by geneticists and pediatricians in our study. While participants greatly believed WGS and precise medicine are the future, a considerable number expressed disquiet about a number of potential risks, including discrimination, psychological stress, and invasion of privacy. One respondent stated his/her full support for the progress of medicine, but explicitly underlined that, as a person, he/she strongly favors patients’ right of autonomy and parents’ power not to consent even if WGS is made part of NBS. Bioethicists have been long exploring the friction between the new genetic and genomic technologies and the principle of respect for autonomy. As these services are becoming more and more routine, this demands a rethinking of traditional interpretations of the concept of informed consent (18). WGS implementation will trigger a more individualized and differentiated set-up for counseling and informed consent. Greater involvement of parents will require development of targeted educational strategies and outreach messages (17).

Potential discrimination was raised as a problem by our survey participants. One geneticist alerted to such dangers and the negative impact on societal acceptance and overall trust in health systems. A number of patient advocates have expressed concerns that clinical use of genetic technologies may reinforce and perpetuate stigmatization and discrimination in both medical and social contexts (19). A pediatrician from our research even pointed out stricter regulations as a prerequisite for a WGS application in NBS. In reality, ethical considerations are likely to dominate this debate, as WGS costs continue to reduce. Economic issues were barely mentioned as a crucial point for WGS implementation in NBS in Bulgaria.

Nevertheless, costs would be a major concern in any country intending to implement WGS use in NBS. Researchers recently evaluated the budget impact of WGS in Germany for all newborns and for diagnostic investigation of tumor patients in different oncologic indications. WGS in NBS was found to lead to costs of € 2.85 billion (an increase of total expenditure by 1.41%). Potential savings in terms of reduced costs for follow-up and improved cost-effectiveness of treatment were not quantified due to the fact that such estimations should be subject of indication-specific evaluations. Even so, this study concluded that WGS has the potential to generate a substantial number of deterministic findings for which treatment options are limited. The authors recommended the selective use of WGS, namely focusing on indications for which WGS has proven medical evidence, and thus saving public health resources (20).

### Prospects for Selective WGS Screening

While there is significant number of objections about non-selective WGS for all newborns, selective WGS screening has been almost unanimously endorsed by previous studies. Cost analysis demonstrates that this procedure could be cost-effective and result in improvements in expectancy and quality of life for affected infants (12). Most recently, experts in genetics, pediatrics, public health, and health policy issued a joint statement, containing a set of recommendations to help inform and guide scientists and clinicians, as well as policy makers regarding the necessary considerations for the use of genome sequencing technologies in NBS. It was stressed that the main objective of NBS should be the targeted analysis and identification of gene variants conferring a high risk of preventable or treatable conditions, for which treatment has to start in the newborn period or in early childhood (21). Any change in the goals of NBS programs should be discussed carefully and represent the best interests of the child (3, 22). Opinions expressed by our study’s participants were consistent with those NBS policy benchmarks. Moreover, both geneticists and pediatricians supported expanding NBS with selective WGS for specific disorders, provided that broad societal consensus is reached and project funding is allocated.

Selective WGS embraces the same concept as NBS: to be predictable, preventive, and personalized. This activity will represent a step-increase in NBS, leading to opportunities for early detection, better management, and effective treatment throughout someone’s life, including late-onset disorders (23). Improved diagnostic capacity is of paramount importance in fields like rare diseases for example. Selective WGS in NBS; this could eliminate the need for an otherwise expensive diagnostic odyssey, by substantially decreasing extra referrals and psychological stress for patients and caregivers (12).

In our study, respondents suggested project funding and cross-border partnerships as optimal settings for launching selective WGS in NBS. European reference networks (ERNs) for rare diseases, which started in 2017, are a recent illustration of such collaborations (24, 25). ERNs are offering, in fact, a unique chance for advanced genetic and genomic approaches. These networks possess sufficient infrastructure and resources to initiate pilot projects to generate evidence on feasibility, efficiency, and cost-effectiveness of selective WGS in NBS. In turn, selective WGS screening could boost significantly rare disease diagnostic capacity at both EU and national levels. As congenital genetic disorders place a disproportionally high burden on families and health systems, selective WGS in NBS could have a major impact in medical practice and public health.

## Conclusion

While non-selective WGS for all newborns is not currently perceived as feasible, pediatricians and geneticists do believe that selective WGS could strengthen current NBS programs. Cross-border project collaborations may set the stage for generating experience and evidence on these complex issues. Further research, including multi-stakeholder partnerships among pediatricians, geneticists, and patients should help guide the development of health policy and practice regarding this concept.

## Ethics Statement

Approval by an Ethics committee was not required for this study. The survey was sociological from a methodological point of view, with no clinical research. No personal data were saved or analyzed.

## Author Contributions

GI and SI contributed to the conception and design of the study, recruitment of survey respondents, analyzing the data, and writing the draft. SW contributed to the conception and design of the study, analyzing the data, and writing the draft. RS contributed to the conception and design of the study, analyzing the data and critical review of the manuscript.

## Conflict of Interest Statement

The authors declare that the research was conducted in the absence of any commercial or financial relationships that could be construed as a potential conflict of interest.
