# Supplementary material for: Whole-Genome Sequencing in Newborn Screening—Attitudes and Opinions of Bulgarian Pediatricians and Geneticists
Source: Front Public Health. 2017 Nov 20;5:308. doi: 10.3389/fpubh.2017.00308 (PMC5715396; doi:10.3389/fpubh.2017.00308)
Supplement: Supplementary file 1 [file data_sheet_1.docx]

**Appendix. Survey questionnaire**

**Part A – Participant’s profile**

1. **Gender:**
2. Male
3. Female
4. **Age in years:**
5. **Highest degree:**
6. MSc
7. PhD
8. DSc
9. **Medical specialty (multiple responses allowed):**
10. **Professional experience in years:**
11. **Main professional sector:**
12. Public
13. Private
14. Equally
15. **Main professional role:**
16. Administration
17. Diagnosis and treatment
18. Research
19. Teaching

**Part II – Participant’s professional attitudes towards WGS in NBS**

1. **How would you assess your knowledge and awareness of NBS in Bulgaria on a 1-5 scale (1 being the least familiar and 5 being the most familiar)?**
2. **How would you assess your knowledge and awareness of WGS on a 1-5 scale (1 being the least familiar and 5 being the most familiar)?**
3. **Do you consider feasible implementing WGS as an adjunct to current NBS in Bulgaria?**
4. Yes
5. No
6. I cannot decide
7. **What is your assessment of the following potential benefits of WGS in NBS on a 1-5 scale (1 being the smallest and 5 being the biggest benefit):**
8. Early diagnosis of the child
9. Early treatment and follow-up of the child
10. Genetic counseling for the family
11. Cost-saving in the long-term
12. Stress-saving in the long-term
13. **What is your assessment of the potential overall benefits of WGS in NBS on a 1-5 scale for the following specific cases/conditions (1 being the smallest and 5 being the biggest benefit):**
14. A newborn carries a mutation that is known to cause a recessive condition
15. A newborn carries a mutation that is known to cause a treatable childhood-onset condition
16. A newborn carries a mutation that is known to cause a non-treatable childhood-onset condition
17. A newborn carries a mutation that is known to cause a treatable adult-onset condition
18. A newborn carries a mutation that is known to cause a non-treatable adult-onset condition
19. A newborn carries genetic markers that are known to increase risk for specific conditions
20. A newborn carries a genetic variant that has unknown phenotype
21. **In case of WGS being implemented as an adjunct to NBS in Bulgaria, what do you think is the most appropriate way to regulate this process?**
22. WGS should be a mandatory part of NBS
23. WGS should be an optional, but highly recommended part of NBS
24. WGS should be available only upon parental request
25. Other:
26. **In case of WGS being implemented as an adjunct to NBS in Bulgaria, what do you think is the most appropriate source to fund this activity?**
27. Public funding
28. Private funding
29. Funding through research projects
30. Funding through research projects and subsequent public funding if justified
31. Other:
32. **In case of WGS being implemented as an adjunct to NBS in Bulgaria, what types of WGS results should be disclosed to parents?**
33. All results should be disclosed to parents
34. Physicians should choose what types of results to disclose to parents
35. Parents should choose what types of results they would like to receive
36. Physicians should choose what types of results to disclose to parents with an option of further decision by the person (the newborn) after attaining legal age
37. Other:
38. **In case of WGS being implemented as an adjunct to NBS in Bulgaria, what do you think is the most appropriate way to regulate additional research with collected and processed anonymized samples?**
39. Samples should not be used for additional research
40. Samples should be used for additional research only with parents’ consent (consent required)
41. Samples should be used for additional research without parents’ consent, but parents can opt out (assumed consent)
42. Samples should be used for additional research without parents’ consent (no consent required)
43. Other:
44. **In case of WGS being implemented apart from NBS in Bulgaria, what is the best time to carry out this activity?**
45. At birth
46. At age of 0-6
47. After attaining legal age
48. In case of specific symptoms
49. Other:

**Part C – Participant’s personal attitudes towards WGS**

1. **Would you consent to your newborn child undergoing WGS?**
2. Yes
3. No
4. I cannot decide
5. **If yes, why?**
6. **If no, why?**
